# The Impact of Artificial Intelligence on Waiting Time for Medical Care in an Urgent Care Service for COVID-19: Single-Center Prospective Study

**DOI:** 10.2196/29012

**Published:** 2022-02-01

**Authors:** Kaio Jia Bin, Adler Araujo Ribeiro Melo, José Guilherme Moraes Franco da Rocha, Renata Pivi de Almeida, Vilson Cobello Junior, Fernando Liebhart Maia, Elizabeth de Faria, Antonio José Pereira, Linamara Rizzo Battistella, Suzane Kioko Ono

**Affiliations:** 1 Hospital das Clinicas Faculdade de Medicina Universidade de Sao Paulo Sao Paulo Brazil; 2 Department of Internal Medicine Federal University of Sao Paulo Sao Paulo Brazil; 3 Escola de Engenharia de Sao Carlos Universidade de Sao Paulo Sao Carlos Brazil; 4 Faculdade de Medicina Universidade de Sao Paulo Sao Paulo Brazil; 5 Department of Gastroenterology Faculdade de Medicina Universidade de Sao Paulo Sao Paulo Brazil

**Keywords:** COVID-19, artificial intelligence, robotic process automation, digital health, health care management, pandemic, waiting time, queue, nonvalue-added activities

## Abstract

**Background:**

To demonstrate the value of implementation of an artificial intelligence solution in health care service, a winning project of the Massachusetts Institute of Technology Hacking Medicine Brazil competition was implemented in an urgent care service for health care professionals at Hospital das Clínicas of the Faculdade de Medicina da Universidade de São Paulo during the COVID-19 pandemic.

**Objective:**

The aim of this study was to determine the impact of implementation of the digital solution in the urgent care service, assessing the reduction of nonvalue-added activities and its effect on the nurses’ time required for screening and the waiting time for patients to receive medical care.

**Methods:**

This was a single-center, comparative, prospective study designed according to the Public Health England guide “Evaluating Digital Products for Health.” A total of 38,042 visits were analyzed over 18 months to determine the impact of implementing the digital solution. Medical care registration, health screening, and waiting time for medical care were compared before and after implementation of the digital solution.

**Results:**

The digital solution automated 92% of medical care registrations. The time for health screening increased by approximately 16% during the implementation and in the first 3 months after the implementation. The waiting time for medical care after automation with the digital solution was reduced by approximately 12 minutes compared with that required for visits without automation. The total time savings in the 12 months after implementation was estimated to be 2508 hours.

**Conclusions:**

The digital solution was able to reduce nonvalue-added activities, without a substantial impact on health screening, and further saved waiting time for medical care in an urgent care service in Brazil during the COVID-19 pandemic.

## Introduction

### Background

Artificial intelligence (AI) has arrived in the field of health care as an aurora, gradually bringing changes and innovations in medical practices. AI-based medical apps and platforms can assist physicians to make better clinical decisions such as in radiology imaging by replacing potentially subjective judgments made by the human eye [1,2]. However, pressures of cost, high expectations, uncertain benefits, a large variety of stakeholders involved, data sharing, and patient safety remain challenging obstacles in the implementation of AI in health care [3-5].

A digital solution was recently developed for the Hospital das Clínicas of the Faculdade de Medicina da Universidade de São Paulo (HCFMUSP) to assist with the reception of patients at the urgent care service. To demonstrate the value of such AI implementation in a health care service, we performed a comparative before-and-after study to understand the impact of the digital solution on the waiting time for medical care [6].

### Electronic Health Records and Waiting Time

Since their initial development in the 1970s [7], the use of electronic health records (EHRs) has become increasingly common in most hospital centers worldwide with advances in the area of information technology [8]. However, use of an EHR is associated with an increase in the time needed to fill out the information [9]. Studies in this area have indicated that doctors spend more time with an EHR system than with direct patient care [10,11].

Despite general satisfaction of doctors with EHR systems [12], the increase in the time spent to use an EHR can contribute to burnout and reduce the quality of the doctor-patient relationship, resulting in a worse interaction [13,14]. Accordingly, several medical universities, especially in the United States, have been seeking strategies to reduce the time spent on EHRs [15], as a prolonged waiting time is one of the most common reasons for a patient to give up on being seen by a doctor in an emergency department [16,17]. The assessment of dissatisfaction is related to a waiting time that is twice as long as that of a fully satisfied patient [18]. Thus, in addition to increasing the patient’s level of satisfaction [19,20], a reduction of waiting time also reduces patient evasion due to fatigue and frustration in the emergency room [17]. 

### The Pandemic

With arrival of the COVID-19 pandemic in Brazil [21], the HCFMUSP established a special operation for their health care professionals with respiratory symptoms at Centro Especializado em Atendimento ao Colaborador (CEAC). Under this system, health care workers with respiratory symptoms are screened quickly to rapidly confirm or dismiss COVID-19 infection. In the first months of the pandemic, the service volume exceeded 3000 visits.

To reduce the length of stay and the face-to-face interaction between symptomatic health care professionals and the CEAC’s administrative team, a digital solution was proposed to be implemented with the primary goal of saving the waiting time to receive medical care.

### Motivation and Aim

To demonstrate the benefits of an AI solution in health care and to reduce the face-to-face interaction during the pandemic, the digital solution was proposed to be implemented. The aim of this study was to determine the impact caused by implementation of the digital solution in the urgent care service for health care professionals of HCFMUSP by assessing the reduction of nonvalue-added activities and its effect on the time required for nurses to screen patients and the waiting time for patients to receive medical care.

### Main Research Questions

We addressed the following questions: Was the digital solution able to automate the medical care registration after nurses perform health screening in the CEAC’s urgent care center? Did use of the digital solution by the health screening team increase the time of screening in CEAC’s emergency department [22]? Did implementation of the AI-based digital solution reduce the waiting time to receive medical care in comparison with attendance without automation at the medical care registration?

## Methods

### Study Design and Setting

This was a single-center, comparative, prospective study that followed the Public Health England guide “Evaluating Digital Products for Health” [6], published in January 2020 by the British government to guide and evaluate the development or implementation of digital products in the field of health. According to the guide, a comparative “before-and-after” study model [23] was applied to evaluate the digital solution using data collected from the Queue Management System module of the CEAC’s EHR system.

### Population and Sample

Administrative data from the EHR between January 2020 and June 2021 at the emergency department of the CEAC were extracted and anonymized, in which each attendance was individualized according to the number of services, which is a unique entry in the EHR system.

A total of 38,042 visits were identified, 98 of which were eliminated due to inconsistent data, resulting in a database of 37,944 visits, which corresponds to 99.74% of all visits received during the study period (Table 1).

For each visit, we checked the EHR for the time record of the following events: medical care registration, nurse health screening, and medical care.

**Table 1:** Exclusion of data per year.

Reason for exclusion	2020 (n=25,578)	2021 (n=12,464)	Total (N=38,042)
System error on the health screening’s end time record, n	0	3	3
Patient leaving before receiving medical care, n	29	2	31
Absence of health screening time record, n	36	4	40
Medical record initiated another day	7	17	24
All reasons, n (%)	72 (0.28)	26 (0.21)	98 (0.26)
Data used for analysis in this work, n (%)	25,506 (99.72)	12,438 (99.76)	37,944 (99.74)

### Study Period

The digital solution was implemented over the month of June in 2020, with repeated tests and follow-up performed by the research team and the information technology team, and the solution has been considered to be 100% functional and operational since July 2020. For the before-and-after study, we considered the preimplementation period from January to May of 2020 and the postimplementation period from July 2020 to June 2021.

### Variables

All variables were obtained through the EHR’s Queue Management System module of the CEAC. Importantly, only released data were collected, meaning data that were released to the EHR system that cannot be changed, edited, or deleted by a user through the EHR itself.

To assess the impact of the digital solution, we analyzed the events, medical care registration, nurse’s health screening, and medical care received for each visit during the study period, grouped by month.

For medical care registration automation, we checked the time record in the EHR: if the time marker was “null,” this indicated that the digital solution filled in the information needed for the registration by robotic process automation (RPA).

**Figure 1:**
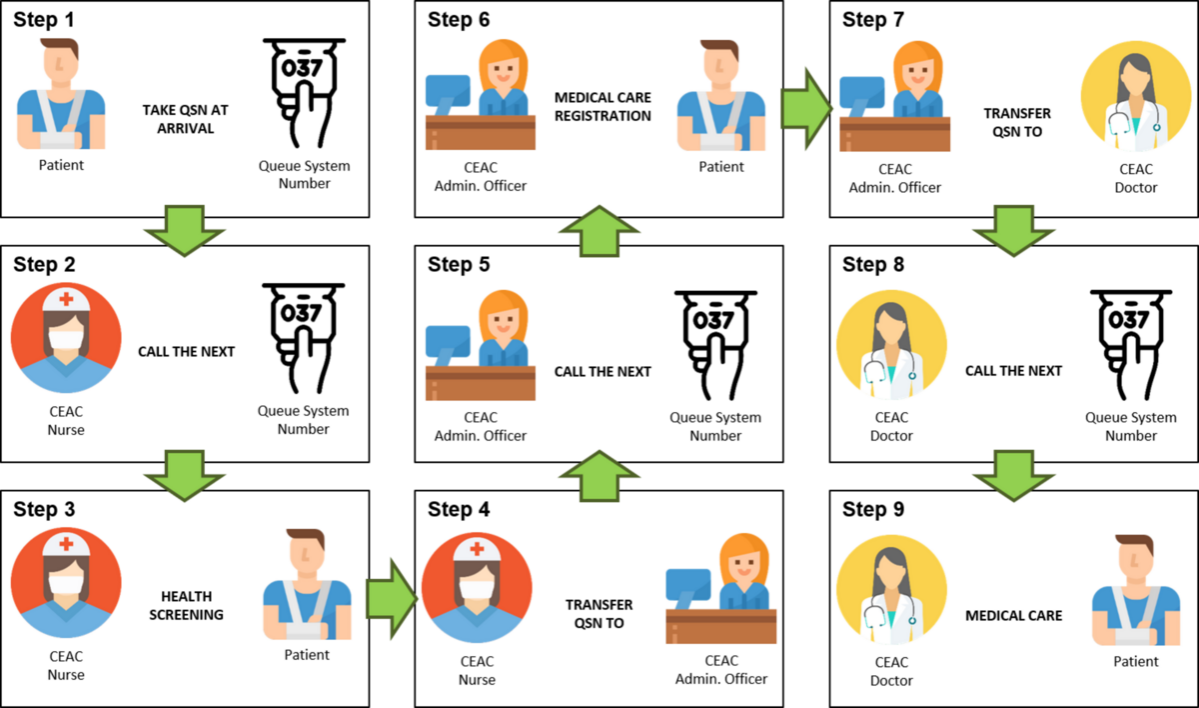
Flowchart before digital solution implementation. Step 1: A patient arrives at the CEAC and takes a queue system number (QSN) for the electronic health record (EHR). Step 2: The CEAC health screening team (nurse) calls the next person in the screening queue. Step 3: The nurse starts the health screening in the EHR. Step 4: At end of the health screening, the nurse transfers the QSN to the registration queue. Step 5: The CEAC administrative team (Admin. Officer) calls the next person in the registration queue. Step 6: The Admin. Officer fills in the medical care registration in the EHR. Step 7: The Admin. Officer transfers the QSN to the medical care queue. Step 8: The CEAC doctor calls the next person in the medical care queue. Step 9: The doctor starts providing medical care. CEAC: Centro Especializado em Atendimento ao Colaborador.

**Figure 2:**
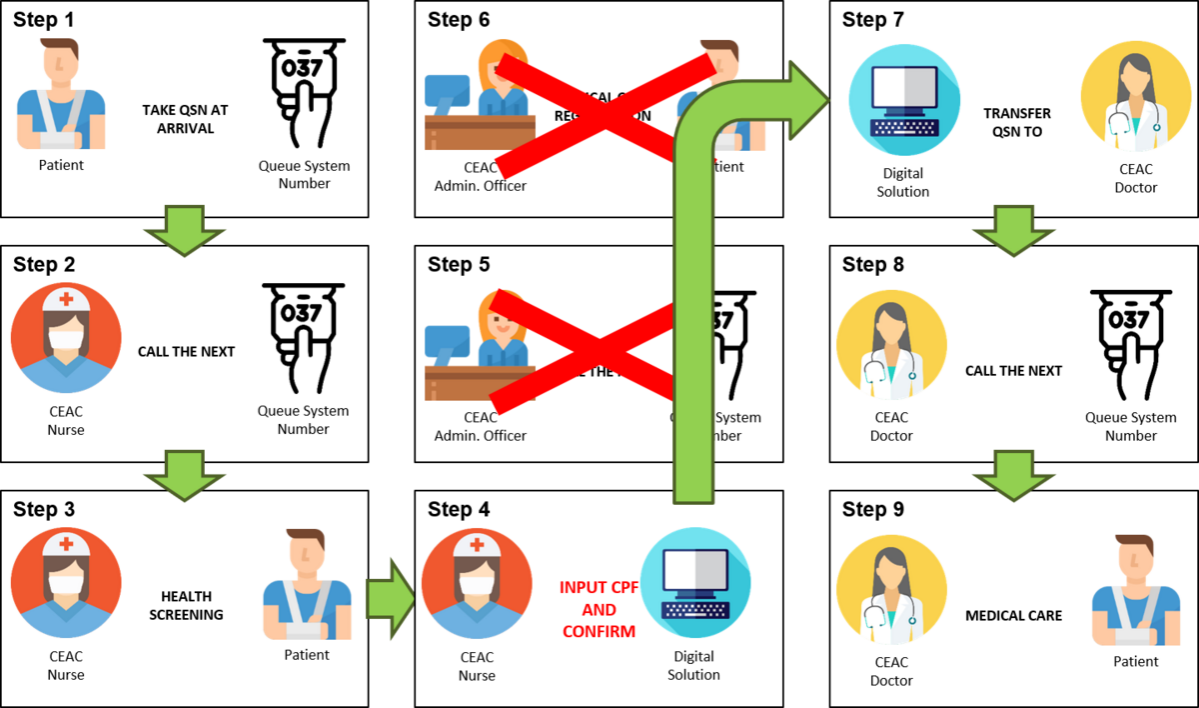
Flowchart after digital solution implementation. Step 1: A patient arrives at the CEAC and takes a queue system number (QSN) for the electronic health record (EHR). Step 2: The CEAC health screening team (nurse) calls the next person in the screening queue. Step 3: The nurse starts the health screening in the EHR. Step 4: At end of the health screening, the nurse inputs the CPF and QSN to the digital solution. Steps 5 to 7: The digital solution fills in the medical care registration in the EHR and transfers the QSN to the medical care queue. Step 8: The CEAC doctor calls the next person in the medical care queue. Step 9: The doctor starts providing medical care. CEAC: Centro Especializado em Atendimento ao Colaborador; CPF: Cadastro de Pessoas Físicas (natural persons number).

A coefficient was created to measure the medical care registration automation, calculated based on the total medical care registration automated divided by the total number of visits per month. The time record for medical care registration in the EHR was checked, which was considered to be the runtime for calculating the time difference between the beginning and end of the registration (see Step 6 of Figure 1 and Figure 2).

The waiting time for registration was used to calculate the time difference between the beginning and end of the registration for the nurse health screening stage (see Steps 3 to 6 of Figure 1 and Figure 2).

The time of screening variable was calculated as the difference in the time for nurses to perform the health screening from the beginning to the end of the screening stage (see Step 3 of Figure 1 and Figure 2).

Waiting time to medical care was based on the time record in the EHR, which was calculated as the time difference between the beginning of the medical care and the end of the nurse’s health screening (Steps 3 to 9 of Figure 1 and Figure 2).

### Data Collection and Analysis

The data were extracted from the EHR and compiled in Microsoft Excel 2010. Each visit was counted per month with Excel’s Pivot Table function. The arithmetic average was used as a measure to assess the data per month.

### The Digital Solution

All activities after the first health screening were identified, and the RPA technology was used to build a software application to replace the tasks of the CEAC’s administrative team before providing medical care. After 8 weeks of development, the digital solution was finally delivered in June 2020 to the CEAC’s health screening team.

Developed for a web platform, the application uses modern Node-JS, Restful, and SOAP [24] technologies to process data entries generated by the nursing team, and automates the administrative processes at the EHR, interpreting data entry without human interaction.

Two key pieces of information were considered essential for this application: (1) the queue system number, which is a key number issued by the CEAC’s EHR for each patient to organize the call order that is printed on paper and displayed on the panel; and (2) the Cadastro de Pessoas Físicas (CPF; translation: Natural Persons Number), which is an 11-digit federal registration number assigned to individual Brazilian taxpayers [25].

At the end of the health screening, the nurse inputs the patient’s CPF to the digital solution, and through the RPA, the digital solution searches for data linked to the entered CPF and fills in the medical care registration form with the necessary information.

As the health screening is completed before the medical care registration, all screening information is linked only to the queue system number. Therefore, with the RPA, the digital solution searches for the screening information linked to the queue system number and makes a definitive link to the patient’s health record after the medical care registration. That is, if the digital solution successfully finds the patient’s profile data in the EHR through the CPF, the subsequent medical care registration process will be automated and the patient will go straight to the next step, which is to be called by the doctor (Step 8 of Figure 2).

The digital solution was installed at the workstation of the nursing team responsible for the health screening, and the entire nursing team of the CEAC was trained to use the system during the month of June.

## Results

### Digital Solution for Medical Care Registration Automation

A total of 37,944 visits over the 18 months between January 2020 and June 2021 were analyzed. We calculated the registration automation coefficient to demonstrate the degree to which the digital solution automated the medical care registration process (Table 2).

With the digital solution, medical care registration has been automated since the month of its implementation (June 2020) in 69% of visits, ranging from a rate of 87% to 95% by month, with an RAC of 92% from July 2020 to June 2021, its first year of implementation (Table 2).

**Table 2:** Distribution of total health screening, total medical care registration (MCR) in person and with automation, and the corresponding registration automation coefficient (RAC).

Period		Total visits, N		MCR with automation, n	MCR with administrative team, n		RAC, %
**Preimplementation**							
	January 2020	1322	0		1322	0	
	February 2020	992	0		992	0	
	March 2020	3361	0		3361	0	
	April 2020	3338	0		3338	0	
	May 2020	2916	0		2916	0	
	Sum	11,929	0		11,929	0	
Implementation: June 2020		2199		1527	672		69
**Postimplementation**							
	July 2020	2143	2043		100	95	
	August 2020	1915	1711		204	89	
	September 2020	1562	1466		96	94	
	October 2020	1512	1396		116	92	
	November 2020	2261	2031		230	90	
	December 2020	1985	1871		114	94	
	January 2021	1743	1640		103	94	
	February 2021	1872	1780		92	95	
	March 2021	2518	2276		242	90	
	April 2021	1780	1645		135	92	
	May 2021	2265	2072		193	91	
	June 2021	2260	1962		298	87	
	Sum	23,816	21,893		1923	92	
Total		37,944		23,420	14,524		—^a^

^a^Not applicable.

### Nurse Health Screening

In the period prior to implementation of the digital solution, the monthly average time of screening from January to May 2020 ranged from 2 minutes and 37 seconds to 3 minutes and 2 seconds, with a mean in this 5-month period of 2 minutes and 54 seconds (Table 3).

During the month of implementation of the digital solution, in June 2020, the mean time of screening was 3 minutes and 21 seconds, representing an increase of 16% compared with the average of the preimplementation period (Table 3). In the postimplementation period, the mean time of screening per month ranged from 2 minutes and 41 seconds to 3 minutes and 23 seconds, with an overall average of 3 minutes for this period (Table 3).

**Table 3:** Distribution by month of total health screenings and average time of screening divided by period and analysis groups.

Period		Health screenings, n	Mean time of screening (minutes:seconds)	Change from the average for the preimplementation period, %	
**Before automation with the digital solution**					
	January 2020	1322	2:58	2	
	February 2020	992	2:54	0	
	March 2020	3361	2:37	–9	
	April 2020	3338	3:01	4	
	May 2020	2916	3:02	5	
	Total	11,929	2:54	N/A^a^	
Implementation: June 2020		2199	3:21	16	
**After automation with the digital solution**					
	July 2020	2143	3:21	16	
	August 2020	1915	3:23	17	
	September 2020	1562	3:14	12	
	October 2020	1512	2:55	1	
	November 2020	2261	2:44	–6	
	December 2020	1985	2:41	–7	
	January 2021	1743	3:03	6	
	February 2021	1872	2:45	–5	
	March 2021	2518	3:01	4	
	April 2021	1780	2:59	3	
	May 2021	2265	2:55	1	
	June 2021	2260	3:02	5	
	Total	23,819	3:00	N/A	
Overall		37,944	2:59	N/A	

^a^N/A: not applicable.

The variation in the arithmetic average of the time of screening from pre- to postimplementation showed an increase ranging from 12% to 17% from June to September 2020, whereas the change for the other months of the postimplementation period ranged from –7% to 5%; thus the change for the 5 months prior to the implementation ranged from –9% to 5% in relation to the average for the period from January to May 2020 (Table 3).

### Waiting Time for Medical Care

With the digital solution, the medical care registration was automated for 92% of visits. This meant that the HCFMUSP patients did not need to wait for the administrative officer to call the queue service number or to wait for the registration procedure. Thus, two nonvalue-added activities were reduced before the final product of receiving medical care (Figure 2).

Table 4 compares the waiting time for medical care with automation of medical care registration through the digital solution and with that performed by an administrative officer.

Compared with the group without automation, the group with automation by the digital solution had a reduction in waiting time ranging from 5 minutes to 12 minutes and 45 seconds, with an average in the postimplementation period (July 2020 to June 2021) of 11 minutes and 51 seconds in waiting for medical care after the nurse’s health screening (Table 4).

**Table 4:** Waiting time for medical care with and without automation from January 2020 to June 2021.

Period			With automation					Without automation					Percent change				Time reduction	
			Visits, n		Mean waiting time (hours:minutes:seconds)		Visits, n			Mean waiting time (hours:minutes:seconds)								
**Before implementation**																		
	January 2020	N/A^a^		N/A		1322			0:45:46		N/A				N/A			
	February 2020	N/A		N/A		992			0:35:01		N/A				N/A			
	March 2020	N/A		N/A		3361			1:15:28		N/A				N/A			
	April 2020	N/A		N/A		3338			0:30:25		N/A				N/A			
	May 2020	N/A		N/A		2916			0:36:10		N/A				N/A			
	Total	N/A		N/A		2386			0:46:36		N/A				N/A			
Implementation: June 2020			1527		0:13:45		672			0:24:24		N/A				N/A		
**After implementation**																		
	July 2020	2043		0:20:12		100			0:31:18		–35				0:11:06			
	August 2020	1711		0:26:58		204			0:39:05		–31				0:12:08			
	September 2020	1466		0:17:33		96			0:26:51		–35				0:09:17			
	October 2020	1396		0:29:12		116			0:38:20		–24				0:09:08			
	November 2020	2031		0:41:18		230			0:52:10		–21				0:10:52			
	December 2020	1871		0:30:48		114			0:37:13		–17				0:06:25			
	January 2021	1640		0:19:28		103			0:24:28		–20				0:05:00			
	February 2021	1780		0:25:14		92			0:31:55		–21				0:06:42			
	March 2021	2276		0:32:32		242			0:39:34		–18				0:07:01			
	April 2021	1645		0:30:17		135			0:37:49		–20%				0:07:32			
	May 2021	2072		0:53:12		193			1:05:57		–19				0:12:45			
	June 2021	1962		0:40:40		298			0:53:07		–23				0:12:27			
	Total	1824		0:31:20		160			0:43:12		–27				0:11:51			

^a^N/A: not applicable.

### Medical Care Registration Before and After Implementing the Digital Solution

Prior to implementation of the digital solution, 100% of the medical care registrations were performed by the administrative team after they were transferred the queue system number by the nursing team (see Steps 4 to 7 in Figure 1).

In this period, the average waiting time for the medical care registration was 4 minutes and 48 seconds and the average runtime for registration was 2 minutes and 4 seconds, for an overall average 6 minutes and 52 seconds spent for the activity (Table 5).

**Table 5:** Arithmetic averages of waiting time and runtime of the medical care registration performed by the administrative team before and after implementation of the digital solution.

Period		Waiting time (minutes:seconds)	Runtime (minutes:seconds)	Total activity time (minutes:seconds)
**Before implementation**				
	January 2020	3:34	1:48	5:22
	February 2020	3:56	1:42	5:38
	March 2020	8:21	2:12	10:32
	April 2020	3:02	2:07	5:09
	May 2020	3:37	2:06	5:43
	Overall mean	4:48	2:04	6:52
**After implementation**				
	July 2020	8:38	3:04	11:42
	August 2020	10:55	2:41	13:36
	September 2020	10:14	3:06	13:20
	October 2020	8:40	3:46	12:26
	November 2020	19:14	2:57	22:11
	December 2020	9:40	3:18	12:58
	January 2021	9:29	3:33	13:02
	February 2021	9:45	3:00	12:45
	March 2021	13:18	3:16	16:33
	April 2021	8:51	3:35	12:26
	May 2021	13:15	3:04	16:19
	June 2021	13:17	2:51	16:08
	Overall mean	12:11	3:07	15:18

With automation by the digital solution, the time taken to perform the medical care registration became null, as the AI by RPA could fill in the data needed for the registration instantly, whereas humans need to manually enter each letter or number by keyboard, in addition to the waiting time for the activity to be executed.

The average waiting time for the start of the registration by the administrative officer (Step 5 of Figure 1) in the postimplementation period of the digital solution was 12 minutes and 11 seconds, whereas the average time for the administrative officer to manually complete the registration was 3 minutes and 7 seconds, representing a total activity time of 15 minutes and 18 seconds (Table 5).

Overall, there was a 154% increase in the waiting time and a 51% increase in the runtime of the medical care registration performed by the administrative team after the digital solution was implemented.

The monthly averages before and after automation with the digital solution are summarized in Table 6. According to these values, the monthly time savings (monthly mean visits×12 months×total activity time for medical registration) realized by adopting the digital solution is estimated at 2507 hours, 42 minutes, and 24 seconds.

**Table 6:** Summary of monthly arithmetic average values before and after implementation of the digital solution with robotic process automation.

Item	Before implementation (January to May 2020)	After implementation (July 2020 to June 2021)	
		Without digital solution	With digital solution
Monthly mean number of visits	2386	160	1826
Mean time of screening (minutes:seconds)	2:54	N/A^a^	3:00
Mean waiting time for MCR^b^	4:48	12:11	N/A
Mean runtime of MCR	2:04	3:07	N/A
Total activity of MCR (waiting+runtime)	6:52	15:18	N/A
Mean waiting time for medical care	46:36	43:12	31:20

^a^N/A: not applicable.

^b^MCR: medical care registration.

## Discussion

### Principal Findings

In this study, we analyzed 99.7% of the visits performed at the CEAC in a period of 18 months (January 2020 to June 2021) spanning the period before, during, and after a digital solution with RPA was implemented (June 2020), which was considered 100% functional as of July 2021.

Before the solution was implemented, all (100%) health care professionals screened by the nursing team had to pass through the administrative team for medical care registration, which is an administrative task aimed at ensuring the appropriate entry of information into the EHR; although this is very important, it is an extra step in the process (Figure 2).

After implementation of the digital solution and over its first 12 months of operation, there was a 92% success rate in medical care registration automation at the end of the health screening by entering the patient’s CPF number in the computer (Table 2). Therefore, this digital solution is effective for the great majority of visits.

As a result, the direct contact of a health care professional presenting to the HCFMUSP with predominantly respiratory symptoms with the CEAC administrative team was reduced, which contributed to maintaining social distancing during the pandemic. This also allowed for relocation of a portion of the administrative team responsible for the medical care registration to other activities, thus increasing the mean waiting time for the medical care registration performed by an administrative officer (Table 5).

The 8% of HCFMUSP health care professionals who needed to be registered by an administrative officer were identified by the local CEAC managers as those with an error in the CPF number in the EHR data profile. As a result, the mean runtime of medical care registration for this group was 51% higher than that of the period prior to implementation of the digital solution (Table 5), based on the need for data correction in the patient’s profile.

This activity of medical care registration performed by an administrative officer is considered to be a nonvalue-added activity according to the lean manufacturing concept [26], because even without this activity, the customer reaches the final product (medical care in this case) and in a shorter time [27,28]. This digital solution with automated medical care registration will therefore help administrative officers to dedicate their time to more important activities with more human contact, compassion, and empathy.

We also found an increase in the overall time taken for the nurse’s health screening with introduction of the digital solution in the first 3 months after implementation, which is consistent with results found in previous studies related to the implementation of electronic record systems in emergency care [22,29]. In the following months, as of October 2020, the mean time of screening demonstrated similar variations to the period prior to implementation, indicating a possible learning curve for the digital solution in the first 3 months (Table 3).

Moreover, with automation of the medical care registration process, the patients had a shorter wait to be seen by the doctor (Table 4). This saving in waiting time is consistent with the total time required for medical care registration performed by the administrative team (Table 5). By eliminating the nonvalue-added activity, the patient gained the time they would otherwise waste with the medical care registration process [26,27].

Considering that the activity involving medical care registration prior to implementation of the solution took an average of 6 minutes and 52 seconds (Table 6) and a monthly average of 1826 visits involved automated registration, the savings time was approximately 209 hours per month or 2508 hours in this 12-month period.

### Limitations

The total time in the patient’s journey was not evaluated in this study, because this could be affected by other influencing factors such as the number of nurses, administrative officers, and doctors on duty. We also did not administer a satisfaction survey to verify whether the savings in waiting time reflected a positive perception for the patient’s experience.

### Cost

Developing this digital solution at the CEAC took 160 hours of labor by a senior programmer, at a total final cost of Brazil real (R$) 16,000 (US $2915.18 based on an exchange rate of US $1.00=R $5.4885 at the time of the study). The application was initially tested on a tablet and then installed on the desktop computer of the health screening room. There were no other expenses, which means that in the 12 months of operation, for each hour saved in waiting time, US $1.16 was spent.

### Conclusions

The year 2020 will always be remembered as the first year of the COVID-19 pandemic. On February 3, 2020, the Ministry of Health of Brazil declared a health emergency of national importance due to human infection by the new coronavirus through Ordinance No. 188 [21], and the following month, the World Health Organization declared a global pandemic [30,31].

In the midst of the pandemic, implementation of the digital solution eliminated the need for an administrative office to register medical care at the CEAC in 92% of cases, thus reducing contact between potentially symptomatic patients and administrative staff.

The introduction of the digital solution in the nursing routine increased the health screening runtime in the first 3 months of use by around 16%, which then returned to the standard of preimplementation after the fourth month of full use.

The waiting time for medical care with automated medical care registration by the digital solution was, on average, almost 12 minutes shorter than that required when automation was not possible. This time saving of around 2500 hours fully justifies the cost and time invested in developing the solution, bringing the prospect of investments in new functionalities for the digital solution.

The same RPA could be applied to other medical activities such as helping the search for exam results or information in the EHR. Such automation can help to reduce the time a doctor must spend in front of the computer, thus providing more time available for human contact between the doctor and patient.

## Abbreviations

AI: artificial intelligence
CEAC: Centro Especializado em Atendimento ao Colaborador
CPF: Cadastro de Pessoas Físicas (Natural Persons Number)
EHR: electronic health record
HCFMUSP: Hospital das Clínicas of The Faculdade de Medicina da Universidade de São Paulo
RPA: robotic process automation
